# A camelid single-domain antibody neutralizes botulinum neurotoxin A by blocking host receptor binding

**DOI:** 10.1038/s41598-017-07457-5

**Published:** 2017-08-07

**Authors:** Guorui Yao, Kwok-ho Lam, Jasmin Weisemann, Lisheng Peng, Nadja Krez, Kay Perry, Charles B. Shoemaker, Min Dong, Andreas Rummel, Rongsheng Jin

**Affiliations:** 1 Department of Physiology and Biophysics, University of California, Irvine, California USA; 2 Institut für Toxikologie, Medizinische Hochschule Hannover, Hannover, Germany; 3 Department of Urology, Boston Children’s Hospital, Department of Microbiology and Immunobiology and Department of Surgery, Harvard Medical School, Boston, Massachusetts USA; 4 NE-CAT and Department of Chemistry and Chemical Biology, Cornell University, Argonne National Laboratory, Argonne, Illinois USA; 5 Department of Infectious Diseases and Global Health, Tufts Clinical and Translational Science Institute, North Grafton, Massachusetts USA

## Abstract

Antibody treatment is currently the only available countermeasure for botulism, a fatal illness caused by flaccid paralysis of muscles due to botulinum neurotoxin (BoNT) intoxication. Among the seven major serotypes of BoNT/A-G, BoNT/A poses the most serious threat to humans because of its high potency and long duration of action. Prior to entering neurons and blocking neurotransmitter release, BoNT/A recognizes motoneurons via a dual-receptor binding process in which it engages both the neuron surface polysialoganglioside (PSG) and synaptic vesicle glycoprotein 2 (SV2). Previously, we identified a potent neutralizing antitoxin against BoNT/A1 termed ciA-C2, derived from a camelid heavy-chain-only antibody (VHH). In this study, we demonstrate that ciA-C2 prevents BoNT/A1 intoxication by inhibiting its binding to neuronal receptor SV2. Furthermore, we determined the crystal structure of ciA-C2 in complex with the receptor-binding domain of BoNT/A1 (H_C_A1) at 1.68 Å resolution. The structure revealed that ciA-C2 partially occupies the SV2-binding site on H_C_A1, causing direct interference of H_C_A1 interaction with both the N-glycan and peptide-moiety of SV2. Interestingly, this neutralization mechanism is similar to that of a monoclonal antibody in clinical trials, despite that ciA-C2 is more than 10-times smaller. Taken together, these results enlighten our understanding of BoNT/A1 interactions with its neuronal receptor, and further demonstrate that inhibiting toxin binding to the host receptor is an efficient countermeasure strategy.

## Introduction

Botulinum neurotoxins (BoNTs) are among the most poisonous natural substances and are categorized as Tier 1 select agents by the Centers for Disease Control and Prevention (CDC). BoNTs cause botulism in humans and other mammals, birds, and fish; and could be misused as biological weapons^1^. BoNTs function as potent proteases, which cleave the neuronal members of soluble *N*-ethylmaleimide sensitive factor attachment protein receptors (SNAREs) complex. As SNAREs are indispensable for the release of acetylcholine at the neuromuscular junctions (NMJs), cleavage of SNAREs by BoNT results in paralysis of muscles^2, 3^. Among the four major human pathogenic BoNTs (BoNT/A, B, E and F), BoNT/A poses the most serious challenge for medical treatment due to its extremely high potency and extraordinary persistence in human patients^4^. Botulism is primarily a consequence of the flaccid paralysis of respiratory muscles, and for patients exposed to BoNT/A, recovery commonly requires several months on a ventilator within an intensive care unit^5^.

To date, there is no small-molecule drug approved for either prevention or post-intoxication treatment of botulism. Antitoxin therapy is currently the only available treatment for botulism patients. The first FDA-approved antitoxin that neutralizes all seven known BoNT serotypes, BAT (Botulism Antitoxin Heptavalent (A, B, C, D, E, F, G) – (Equine)), is able to neutralize circulating BoNTs thereby preventing further disease progression. However, BAT consists of equine-derived polyclonal IgG antibodies largely processed to F(ab)2, and its use can cause various side effects^6^. An Investigational New Drug (IND) XOMA 3AB, which contains three human monoclonal antibodies, is effective in neutralizing BoNT/A^7^. However, monoclonal antibody drugs are generally expensive, require intravenous administration, and have limited shelf lives. Therefore, alternative therapeutic antitoxin approaches that reduce costs and improve convenience remain an important goal.

Recently, we and other laboratories found that the antigen-binding region (V_H_) of the heavy-chain-only antibodies (VHHs, also referred to as nanobodies) produced by camelids show strong anti-BoNT activities in animal models^8–10^. VHHs possess full antigen-binding capacity with high affinity and specificity, and are advantageous in their small size, remarkable stability, low immunogenicity to humans, as well as ease of production^11^. Furthermore, VHHs usually present convex paratopes that allow improved opportunities to identify agents that bind otherwise inaccessible conformational epitopes, which often represent enzyme active sites and receptor-binding domains^12, 13^. Thus VHHs have excellent promise as components of improved anti-BoNT therapeutic agents.

**Figure 1:**
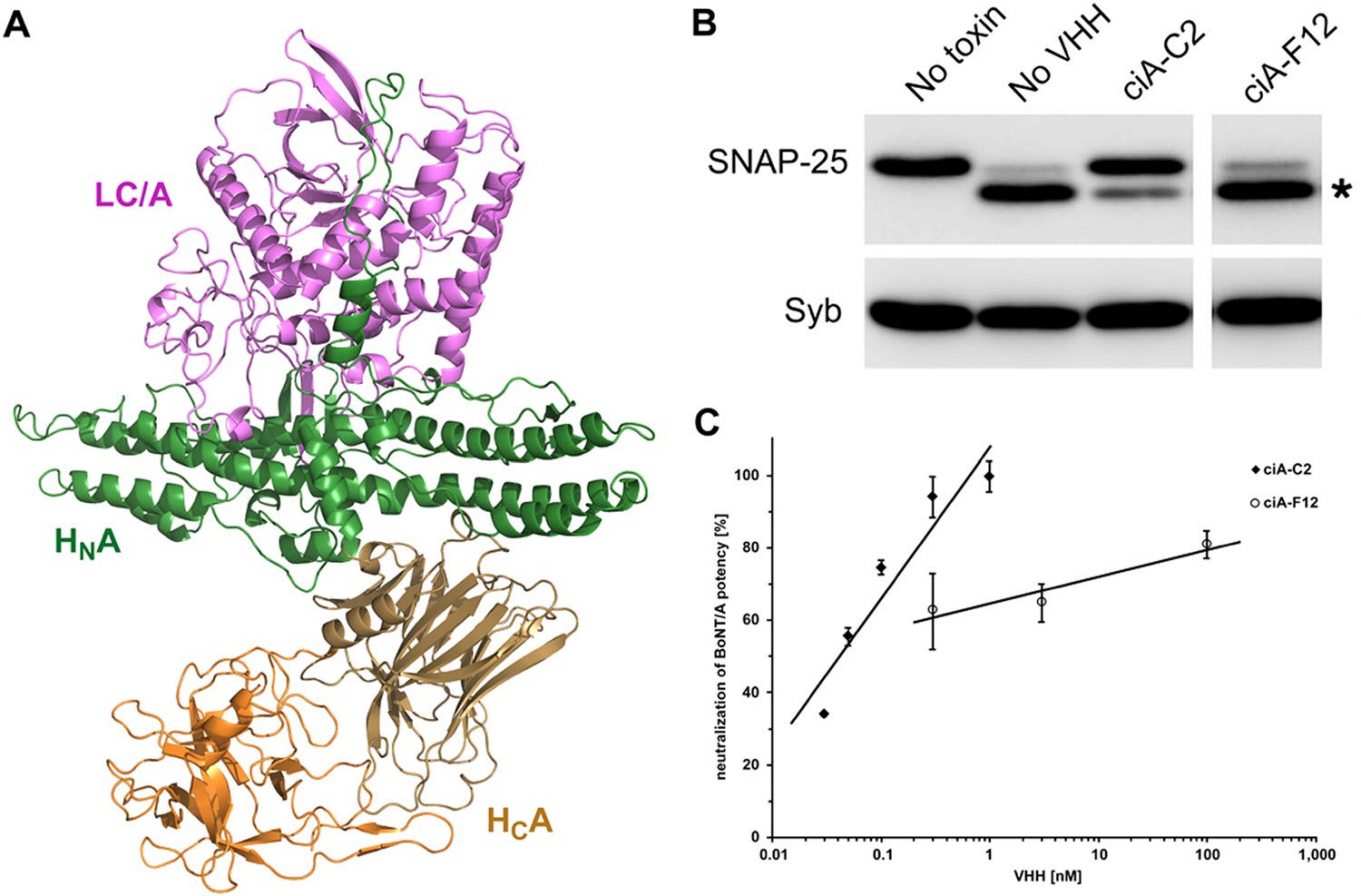
ciA-C2 neutralizes BoNT/A1. (**A**) Overall architecture of BoNT/A1 (PDB: 3BTA). The light chain (LC/A), the translocation domain (H_N_A), and the receptor-binding domain (H_C_A) are colored violet, green, and sand/orange, respectively. (**B**) ciA-C2 inhibits SNAP-25 cleavage in cultured primary neurons. BoNT/A1 (2.5 nM) was pre-incubated with indicated VHHs (50 nM) for 40 min at 4 °C and the mixtures were added to neuron culture media for 7 hrs. Neurons were then harvested and the cell lysates were subjected to immunoblot analysis to examine SNAP-25 and Synaptobrevin (Syb). Cleavage of SNAP-25 by BoNT/A1 generates a smaller fragment, which is indicated by an asterisk. Syb serves as an internal control. (**C**) The neurotoxicity of BoNT/A1 (1.63 pM) in the presence of various concentrations of ciA-C2 or ciA-F12 was assessed in the MPN hemidiaphragm assay with 3–5 technical replicates.

In this study, we focused on an alpaca VHH (named ciA-C2) that potently neutralizes BoNT/A1, one of the eight known BoNT/A subtypes (A1-A8)^10^. To further characterize ciA-C2 and understand the molecular basis of BoNT/A1 neutralization, we mapped the ciA-C2-binding region to the C-terminal receptor-binding domain of BoNT/A1 (H_C_A1) (Fig. 1A) using both a neuron surface binding assay and biochemical binding assays. The extreme toxicity of BoNT/A1 depends to a large degree on its highly specific recognition of motoneurons in NMJs by H_C_A1^14–19^. We determined a high-resolution co-crystal structure of H_C_A1 in complex with ciA-C2, and found that ciA-C2 occupies a strategic site on BoNT/A1 that is needed for the toxin to recognize its receptor SV2, therefore preventing BoNT/A1 from attaching to motoneurons.

## Results

### ciA-C2 potently neutralizes BoNT/A1

In earlier studies, we found that ciA-C2 binds tightly to BoNT/A1 (dissociation constant, K_d_, ~3.7 nM)^10^. We further examined ciA-C2′s neuroprotective capability using a cell based assay. Here, rat primary neurons were treated with BoNT/A1 in the presence of ciA-C2 or ciA-F12, a non-neutralizing BoNT/A1-binding VHH as a control^10^. When tested at a molar ratio of ~1:20 (toxin:VHH), ciA-C2 significantly inhibited the SNAP-25 cleavage by BoNT/A1, while ciA-F12 virtually showed no effect (Fig. 1B). A higher dose of ciA-C2 (~1:100) was needed to achieve a complete inhibition^10^.

To further investigate the biological activities of ciA-C2 against BoNT/A1 at the motor nerve terminals—the site-of-action for all BoNTs—we carried out an *ex vivo* mouse phrenic nerve (MPN) hemidiaphragm assay, a method that closely reproduces *in vivo* respiratory failure caused by BoNT intoxication^14^. As shown in Fig. 1C, pre-incubation with 1 nM of ciA-C2 displayed almost complete neutralization of 1.63 pM BoNT/A1. ciA-F12, which binds to BoNT/A1 with an affinity (K_d_ ~0.24 nM) higher than ciA-C2, showed weak inhibition of BoNT/A1 (1.63 pM) even at a high concentration up to 100 nM. These findings confirmed that ciA-C2 is a potent antitoxin against BoNT/A1, despite its small size that is less than 10% of a conventional IgG.

### ciA-C2 inhibits cell-surface binding of BoNT/A1

In general, neutralization of BoNTs can be achieved by interfering with at least one of the four major steps required for successful nerve cell intoxication. These steps are: (1) BoNT binds to cell surface receptors on motoneurons; (2) BoNT is internalized by receptor-mediated endocytosis; (3) the protease domain of BoNT (light chain, LC) translocates across vesicle membrane to reach the cytosol; and (4) the LC specifically cleaves SNARE proteins^20^. We performed an *in vitro* cleavage assay using the recombinant LC/A with the recombinant SNAP-25 as a substrate, and found that neither ciA-C2 nor ciA-F12 could protect SNAP-25 from being cleaved by LC/A (Fig. S1). This is consistent with our earlier studies suggesting that ciA-C2 binds to H_C_A1^10^. Therefore, we suspected that ciA-C2 may interfere with H_C_A1-mediated receptor binding.

**Figure 2:**
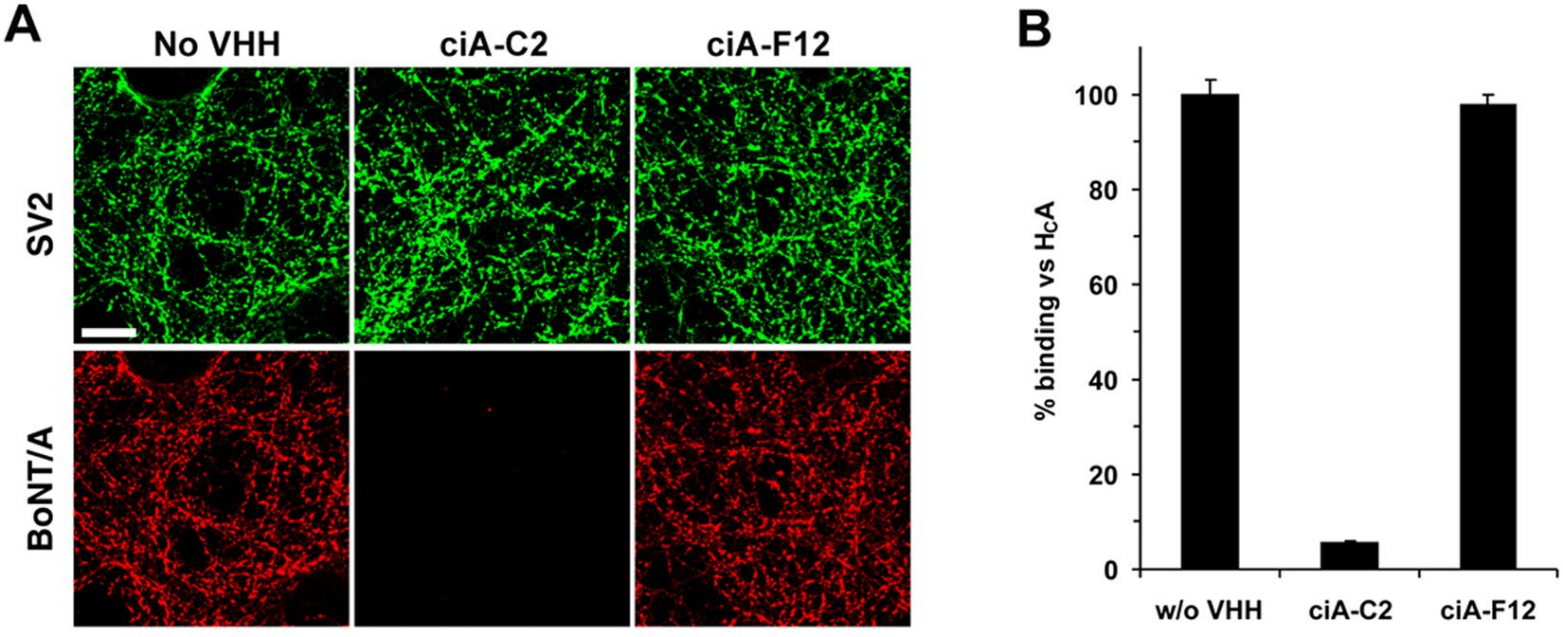
ciA-C2 prevents BoNT/A1 from binding to neurons. (**A**) ciA-C2 blocks the binding of BoNT/A1 to neuronal surface. BoNT/A1 (20 nM) was pre-incubated with indicated VHHs (200 nM) in high K^+^ buffer. The mixtures were used to incubate cultured rat hippocampal neurons for 5 min. Neurons were then washed, fixed, and subjected to immunostaining analysis detecting BoNT/A1. SV2 was detected as a marker for pre-synaptic terminals. Scale bar: 20 μm. (**B**) ciA-C2 blocks synaptosome binding of H_C_A1. Rat brain synaptosomes were incubated with radiolabeled H_C_A together with ciA-C2 or ciA-F12 (200 nM). Synaptosomes were then collected by centrifugation, washed, and subjected to SDS-PAGE and autoradiography. Amounts of bound H_C_A1 were compared with the binding efficiency of the reference H_C_A1 without VHH.

We then carried out two complementary assays to test this hypothesis. First, we examined BoNT/A1 binding to the cultured rat hippocampal neurons, and found that pre-incubation with ciA-C2 almost completely abolished neuronal binding of BoNT/A1, whereas ciA-F12 did not (Fig. 2A). Second, we conducted a synaptosome binding assay using ^35^S radiolabeled H_C_A1 on freshly prepared rat brain synaptosomes. ciA-C2, but not ciA-F12, drastically reduced the binding of H_C_A1 to synaptosomes (Fig. 2B). These findings strongly suggest that ciA-C2 prevents BoNT/A1 from attaching to motor nerve terminals by interfering with its receptor binding.

### High-resolution crystal structure of ciA-C2 in complex with H_C_A1

**Figure 3:**
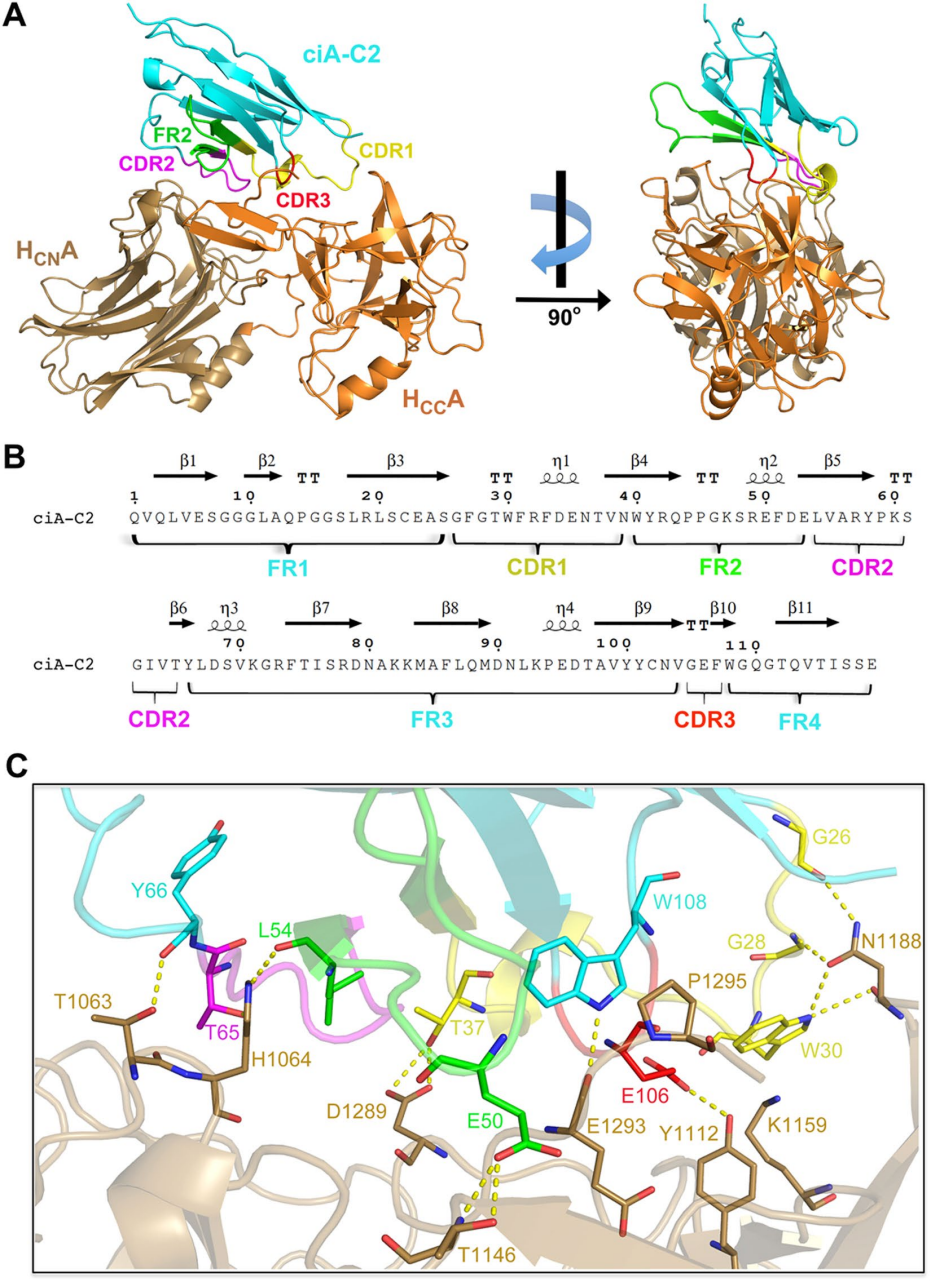
Structure of the H_C_A1-ciA-C2 complex. (**A**) H_C_A1 is colored as in Fig. 1A. ciA-C2 is colored in cyan while its CDR1, CDR2, CDR3 and FR2 regions are colored in yellow, magenta, red and green, respectively. (**B**) Amino acid sequence of ciA-C2 with secondary structures shown on the top (ENDscript 2.0)^57^, and the Kabat complementarity determining regions (CDRs) and framework regions (FRs) indicated on the bottom. (**C**) A close-up view of the interface between ciA-C2 and H_C_A1 using the same color scheme as panel A. Interacting residues are shown in sticks. Yellow dashed lines indicate hydrogen bonds.

In order to understand the molecular details underlying BoNT/A1 neutralization by ciA-C2, we performed co-crystallization and determined the structure of ciA-C2 in complex with H_C_A1 at 1.68 Å resolution (Table 1). There is one ciA-C2-H_C_A1 complex in an asymmetric unit (AU) in the crystal with a 1:1 stoichiometry (Fig. 3A). Binding of ciA-C2 does not cause obvious conformational change to H_C_A1 when compared to H_C_A1 in its apo state (PDB: 3FUO) (root-mean-square﻿ (RMS) deviation of ~0.35 Å over 367 aligned Cα pairs, calculated by Pymol)^21^. ciA-C2 consists of a typical single immunoglobulin domain that contains three complementarity-determining regions (CDRs) and four framework regions (FRs) (Fig. 3A,B). A canonical disulfide bond is formed between residues C22 of FR1 and C102 of FR3.

**Table 1:** Data collection and refinement statistics.

	H_C_A1-ciA-C2 (PDB: 5L21)
**Data collection**	
Space group	P 1 21 1
Cell dimensions	
*a*, *b*, *c* (Å)	50.10 Å; 103.72 Å; 64.70 Å
*α, β, γ* (°)	90.00°; 93.78°; 90.00°
Resolution (Å)	35.99–1.68 (1.71–1.68)^a^
*R*_meas_	0.057 (0.582)
*I/σ*(*I*)	12.8 (2.3)
*CC*_1/2_	0.998 (0.843)
Completeness (%)	99.5 (99.7)
Redundancy	2.9 (2.9)
**Refinement**	
Resolution (Å)	35.99–1.68 (1.74–1.68)
No. reflections	74580
*R*_work_/*R*_free_	0.193/0.205
No. atoms	
Protein	544
Ligand	—
Water	566
*B* factors	
Protein	35.5
Ligand	—
Water	43.1
R.m.s. deviations	
Bond lengths (Å)	0.005
Bond angles (°)	0.096

^a^Values in parentheses are for the highest-resolution shell.

H_C_A1 could be further divided into two subdomains: the N-terminal lectin-like domain (H_CN_A1) and the C-terminal β-trefoil domain (H_CC_A1) (Fig. 3A). H_CC_A1 is a cell-surface binding mediator to both PSG and SV2. The role of H_CN_A1 during toxin uptake is not fully understood, but it is involved in binding of an N-linked glycan on SV2 that strengthens neuron recognition of BoNT/A1^17^. Interestingly, ciA-C2 binds to H_C_A1 at an area located between H_CN_A1 and H_CC_A1. The binding of ciA-C2 bridges the two subdomains, resulting in a buried interface area of 1,150 Å^2^ (calculated by PDBePISA v1.51)^22^. Most of the ciA-C2-H_C_A1 interactions are contributed by residues in the CDR1, FR2, and CDR2 regions of ciA-C2, with few residues in the FR3, CDR3 and FR4 regions (Fig. 3C and Table S1). Residues G26, G28, W30, and T37 of CDR1 form multiple hydrogen bonds with N1188 and D1289 in H_CC_A1. A strong cation-π interaction is observed between W30 of ciA-C2 and K1159 of H_C_A1. T65 of CDR2 and Y66 of FR3 interact with H_CN_A1 by forming hydrogen bonds with H1064 and T1063, respectively. FR2 of ciA-C2 binds both subdomains of H_C_A1: E50 forms two hydrogen bonds with T1146 of H_CC_A1, while L54 forms a hydrogen bond with H1064 of H_CN_A1. In CDR3 and FR4 of ciA-C2, residue E106 interacts with Y1112 of H_CC_A1, while W108 forms a hydrogen bond with E1293 and an aromatic-proline interaction with P1295 at the C-terminus of the toxin.

### ciA-C2 prevents BoNT/A1 from binding to both the peptide and carbohydrate moieties of SV2C

**Figure 4:**
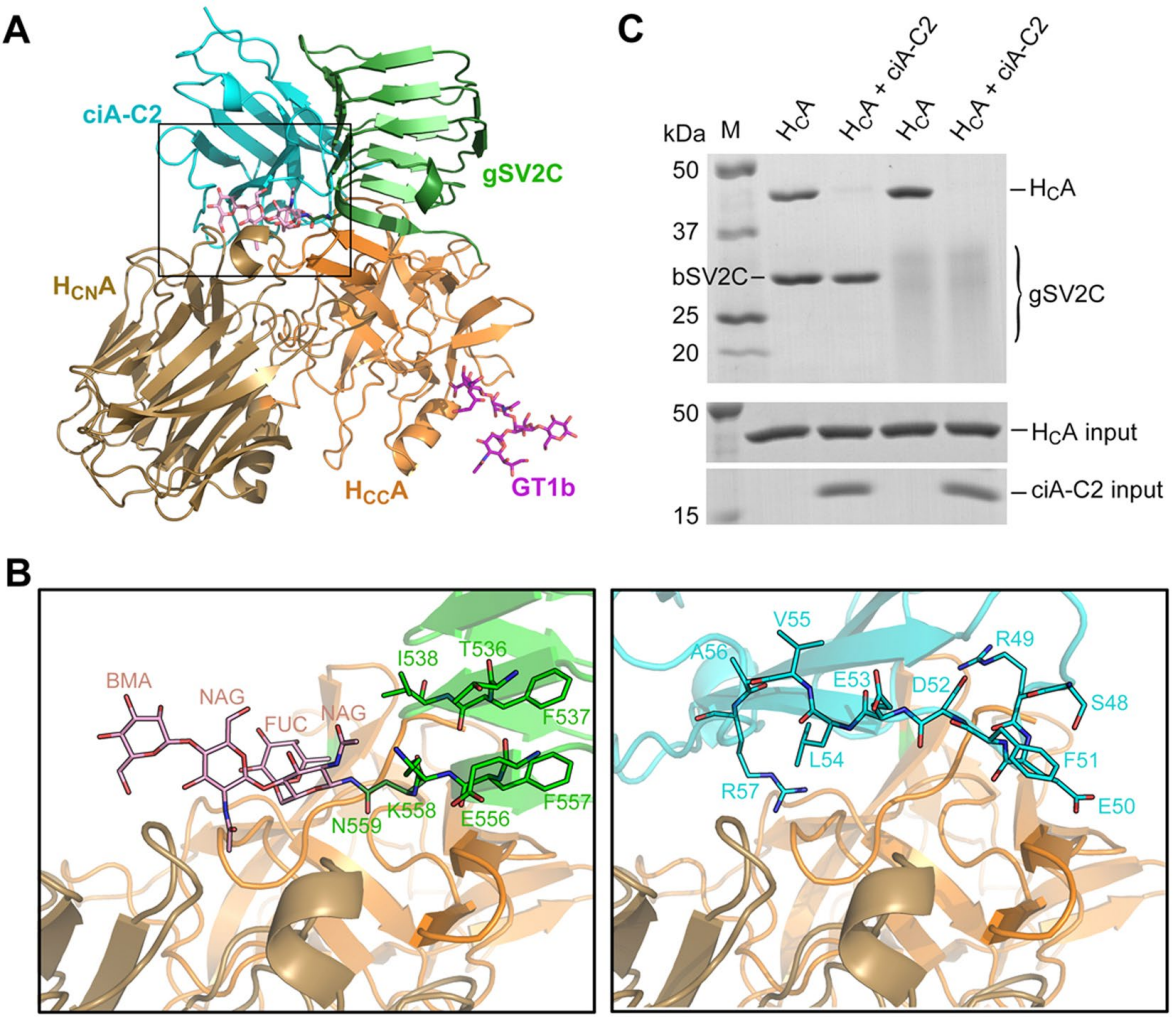
ciA-C2 blocks BoNT/A1 binding to SV2. (**A**) Superposition of the structures of H_C_A1-ciA-C2, H_C_A1-gSV2C (PDB: 5JLV), and H_C_A1-GT1b (PDB: 2VU9) complexes. H_C_A1 and ciA-C2 are colored as in Fig. 3. gSV2C is colored in green while its N559 glycan is shown as pink sticks. GT1b is shown as purple sticks. Close-up views of the interface highlighted in the box is shown in (**B**), where the structures of H_C_A1-gSV2C and H_C_A1-ciA-C2 are shown in the left and the right panel, respectively. T536-T538, E556-N559 and the N559 glycan of gSV2C and the ciA-C2 residues S48-R57 that occupy the same space on H_C_A1 surface are shown as sticks. (**C**) ciA-C2 prevents H_C_A1 from binding to SV2C independent of its glycosylation state. Pull-down assay was performed using H_C_A1 as prey and the His-tagged bSV2C or gSV2C as baits. After binding, the Ni-NTA resins were washed twice, and the bound proteins were released from the resins and subjected to SDS-PAGE for visualization.

It is well accepted that all BoNTs bind to polysialogangliosides and most of them further bind specific protein receptors on the neuronal surface in order to achieve high binding affinity and specificity^16, 19, 23^. Failure of binding to either receptor results in drastic toxicity loss, as demonstrated in many studies^14, 16, 18, 24–27^. A structural comparison with the GT1b-bound H_C_A1 (PDB: 2VU9)^28^ clearly shows that ciA-C2 binds H_C_A1 at a position far from the GT1b-docking site, thus it is unlikely to affect BoNT/A-PSG interaction (Fig. 4A).

We then focused on the BoNT/A1-SV2 interactions. Our recent co-crystal structure of H_C_A1 in complex with the glycosylated SV2C (gSV2C) revealed extensive interactions between them, which are crucial for BoNT/A1’s high neuron tropism^17^. Besides the conventional protein-protein interactions, BoNT/A1 recognizes an N-linked glycan on SV2 that is conserved across vertebrates. A structural comparison between the H_C_A1-ciA-C2 and H_C_A1-gSV2C complexes showed that ciA-C2 largely occupies the binding-site for the SV2C glycan (Fig. 4A). The side chain of ciA-C2-L54 clashes with the aromatic ring of the second N-acetylglucosamine (NAG), while the main chain of ciA-C2-R57 overlaps with the beta-D-Mannose (BMA). Furthermore, the two hydrogen bonds formed between ciA-C2-E50 and H_C_A1-T1146 brought the FR2 loop of ciA-C2 up-close to the toxin, resulting in spatial conflicts with two β–sheets of gSV2C including residues T536-T538 and E556-K558 (Fig. 4B). Therefore, ciA-C2 simultaneously inhibits both the protein- and glycan-based interactions between BoNT/A1 and SV2C. These findings were further supported by an *in vitro* pull-down assay using H_C_A1 as a prey and bSV2C (non-glycosylated SV2C) and gSV2C as baits. ciA-C2 clearly eliminated H_C_A1-SV2C interactions regardless of SV2C’s glycosylation state (Fig. 4C).

### The neutralization mechanism of ciA-C2 is similar to that of a monoclonal antibody in clinical trials

**Figure 5:**
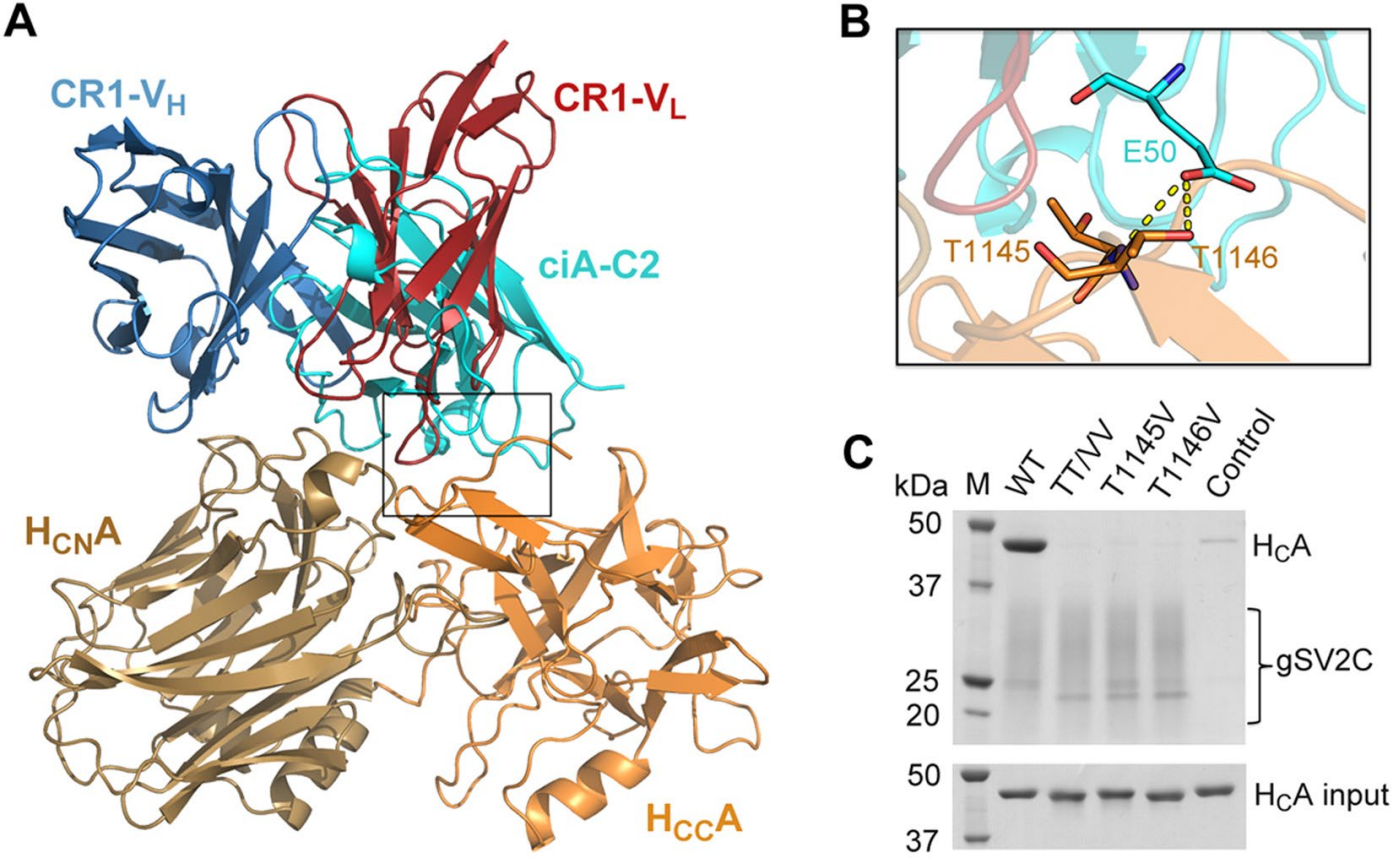
ciA-C2 binds to both subdomains of H_C_A1 while CR1 primarily binds to H_CN_A1. (**A**) Superposition of the structures of the H_C_A1-ciA-C2 and H_C_A1-CR1 (PDB: 2NYY) complexes. (**B**) A close-up view of the interface around the H_CC_A1 subdomain highlighted in a box in panel A. ciA-C2 (cyan) occupies a crucial SV2-binding area on H_CC_A1 (orange) involving residues T1145 and T1146, but CR1 (red) does not. (**C**) Pull-down assays using gSV2C as bait and H_C_A1 (WT and mutants) as preys. TT/VV: H_C_A1-T1145V-T1146V. Ni-NTA resins incubated with H_C_A1-WT in the absence of gSV2C were used as a negative control.

We and other groups have shown that a potent BoNT/A-neutralizing human antibody currently in clinical trials, CR1/CR2, blocks BoNT/A-SV2 recognition^16, 17, 29^. The crystal structure of BoNT/A1 in complex with CR1-Fab (fragment antigen-binding) revealed two separated binding regions mediated by the variable regions in the heavy chain (V_H_) and the light chain (V_L_). We found that ciA-C2 almost completely occupies the V_L_-binding site on H_C_A1 (Fig. 5A). Surprisingly, even though ciA-C2 is only 1/4 size of CR1-Fab (and <10% of CR1-IgG), it occupies a binding interface as big as that of CR1 (1,126 Å^2^ for CR1 vs. 1,150 Å^2^ for ciA-C2). Moreover, ciA-C2 binds to both H_CN_A1 and H_CC_A1, while CR1 mostly targets H_CN_A1 (Fig. 5B).

While CR1 only shows side-to-side clashes with the peptide portion of SV2C, residue E50 of ciA-C2 directly binds to a key SV2-binding residue T1146 in H_C_A1 (Fig. 5B), which forms three hydrogen bonds with F557 of human SV2C and two of them are formed via the atom OG1 of T1146^17^. Earlier studies showed that a T1145A-T1146A mutation almost completely blocked the binding between BoNT/A1 and SV2C^15, 17^. However, we suspected that mutating threonine, a strong sheet-forming residue, to an alanine may cause artificial local secondary structure changes on H_C_A1. Therefore, we designed a new H_C_A1 mutant, T1145V-T1146V, in which Thr-OG1 was replaced with Val-CG1 to abolish OG1-mediated interactions, while both threonine and valine are strong sheet formers. The wild-type and mutations of H_C_A1 were then subjected to a pull-down assay employing gSV2C (Fig. 5C) and bSV2C (data not shown) as baits. We found that even a single atom change in T1145 and T1146 was sufficient to abolish the H_C_A1-SV2C interaction, indicating this area of toxin is extremely important for receptor recognition and highly sensitive to even subtle conformational changes. Both T1145 and T1146 are strictly conserved in all eight known BoNT/A subtypes^30^. Thus, ciA-C2 neutralizes BoNT/A1 by targeting one of the most critical spots on the toxin-receptor interface, which was not observed for CR1/CR2.

### BoNT/HA and various BoNT/A subtypes respond differently to ciA-C2 due to subtle residue changes

We next examined whether ciA-C2 could also recognize other natural variants of BoNT/A1, as BoNT genes are actively evolved and at least 40 different subtypes of BoNT have been reported^31^. We first analyzed a newly reported mosaic toxin type HA (BoNT/HA, also known as BoNT/FA or BoNT/H), which has a hybrid-like structure including a BoNT/A1-like H_C_^32–36^. Amino acid sequence alignments showed that the H_C_ of BoNT/HA (H_C_HA) is ~84% identical to H_C_A1. The overall structure of H_C_HA and H_C_A1 are very similar with a RMS deviation of 0.526 Å over 341 Cα atoms^37^. Some BoNT/A-neutralizing antibodies that target the H_C_ domain were able to neutralize this new toxin^38, 39^. For example, a monoclonal antibody RAZ1 showed an almost identical binding affinity to BoNT/A1 and BoNT/HA. However, antibody CR1/CR2 showed a drastic ~540-fold decreased binding affinity for BoNT/HA when compared to BoNT/A1. In earlier studies, we found that residue N954 and residues S955-S957 of the 3/10-helix of H_C_A1 form multiple hydrogen bonds with CR1/CR2 (through residues N96 and E97 of CR1), while these polar contacts are missing in H_C_HA that is very different from H_C_A1 in this region^37, 38^. Furthermore, an important salt bridge between H_C_A1-R1294 and CR1-D30 is missing in H_C_HA due to an arginine to serine replacement (H_C_HA-S1286)^37^. Therefore, subtle residue changes on the toxin could lead to substantially different responses to the available antitoxins.

**Figure 6:**
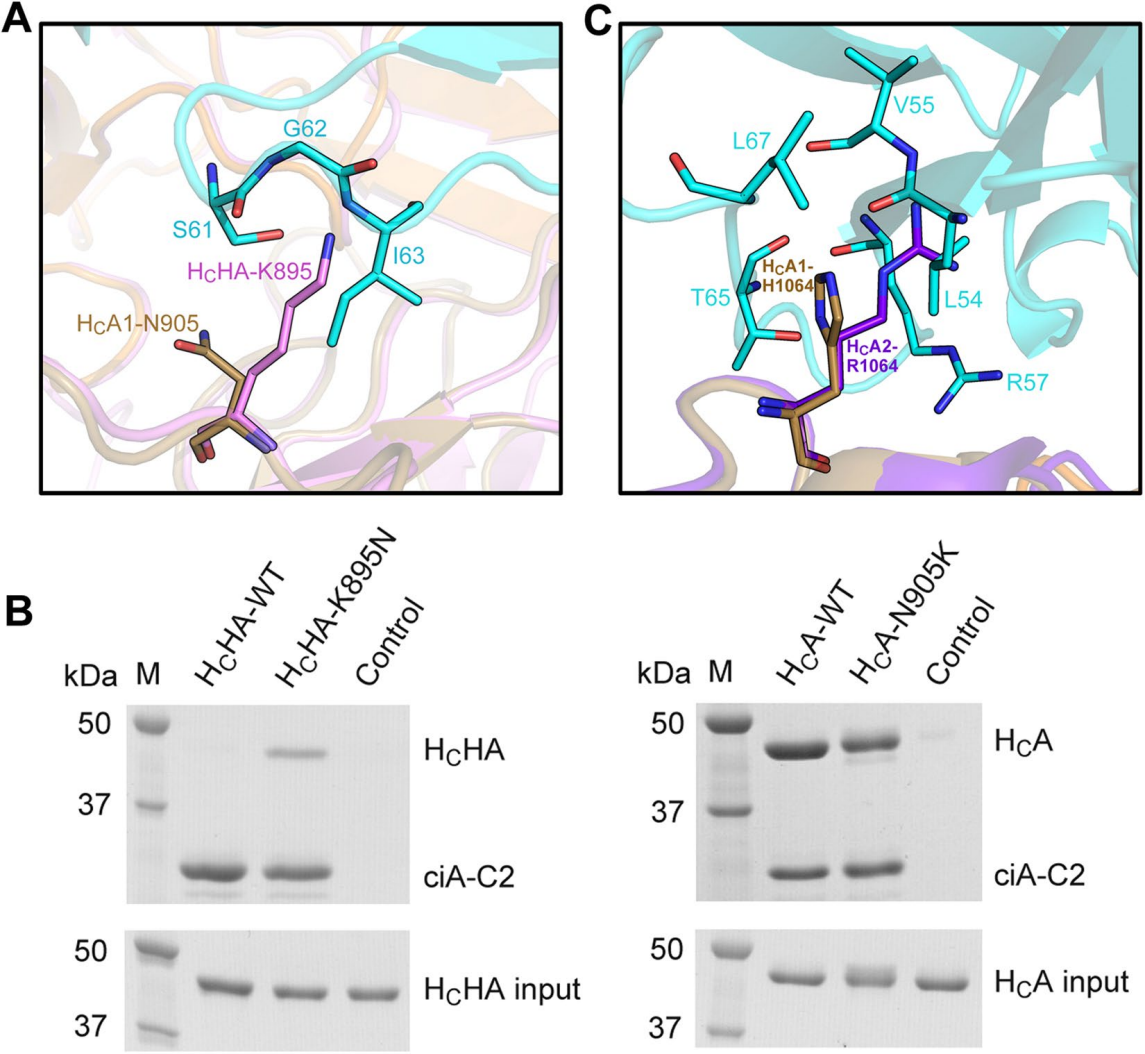
Subtle amino acid differences between BoNT/A1 and BoNT/HA or BoNT/A2 cause different responses to ciA-C2. (**A**) A close-up view showing potential clashes between H_C_HA-K895 and the CDR2 region of ciA-C2. The structure of H_C_HA (PDB: 5V38, colored purple) is superimposed onto the H_C_A1-ciA-C2 complex (H_C_A1: sand; ciA-C2: cyan). (**B**) Pull-down assays using ciA-C2 as a bait and H_C_A1 and H_C_HA variants as preys. Ni-NTA resins incubated with H_C_HA-WT (left panel) and H_C_A1-WT (right panel) in the absence of ciA-C2 were used as negative controls. (**C**) A close-up view at the H_C_A1-H1064-binding pocket on ciA-C2, where H_C_A2-R1064 cannot be accommodated. Structural superposition was made using the structures of H_C_A2 (PDB: 5MOY, colored in deep purple) and the H_C_A1-ciA-C2 complex (H_C_A1: sand; ciA-C2: cyan).

Sequence analyses showed that most of the ciA-C2-binding residues are conserved in BoNT/HA (Fig. S2)^17^. Nonetheless, ciA-C2 did not bind H_C_HA based on a pull-down assay and is apparently not able to neutralize BoNT/HA (Fig. 6B). Taking advantage of the structures of H_C_HA (PDB 5V38)^37^ and the ciA-C2-H_C_A1 complex, we observed a potential clash and a repulsive charge interaction between H_C_HA-K895 and the CDR2 loop (K60-I63) of ciA-C2, whereas the equivalent H_C_A1-N905 did not (Fig. 6A). Can this unique amino acid change in BoNT/HA contribute to the inactivation of ciA-C2? We then designed two single-point mutants, H_C_HA-K895N and H_C_A1-N905K, where the two amino acids were swapped between the two toxins. The pull down assay demonstrated that ciA-C2 was able to recognize H_C_HA-K895N, whereas it displayed a decreased binding to H_C_A1-N905K by ~30% (n = 3), thus confirming our hypothesis (Fig. 6B).

Eight BoNT/A subtypes, termed BoNT/A1-A8, have been identified. The crystal structure of H_C_A2 in complex with the non-glycosylated SV2C-L4 (PDB 5MOY) was reported recently^40^. Structural comparison between H_C_A1 and H_C_A2 suggested that a replacement of H_C_A1-H1064 by R1064 in H_C_A2 may cause a decreased binding to ciA-C2, because H_C_A2-R1064 does not fit well into the H_C_A1-H1064-binding pocket and it clashes with L54 of ciA-C2 and may also repel a neighboring R57 in ciA-C2 (Fig. 6C). Sequence analyses among BoNT/A1-A8 (Fig. S2) showed that BoNT/A3, A6, and A8 also have a H_C_A2-like arginine at this position, thus likely weakens their binding to ciA-C2 as well. Furthermore, the replacement of a ciA-C2-interacting P1295 in BoNT/A1 with S1295 in BoNT/A2 (also exists in BoNT/A3, A7, and A8) may further weaken the binding to ciA-C2. Therefore, the structure of H_C_A2 further confirms the notion that BoNTs could potentially escape from the existing antitoxins via subtle residue changes^33^.

## Discussion

BoNTs are large proteins that induce a wide variety of antibodies that recognize them at different sites and may or may not be able to neutralize the toxins. Even the neutralizing antibodies display vastly different potencies, largely dependent on their neutralizing mechanisms and their affinities. Therefore, a comprehensive understanding of the mechanisms for neutralizing antibodies is urgently needed for further therapeutic development. In this study we present the first high-resolution crystal structure of a BoNT/A1-neutralizing VHH, ciA-C2, in complex with the receptor-binding domain of the toxin. The strong neutralizing activity of ciA-C2 against BoNT/A1 was clearly demonstrated by the *ex vivo* MPN hemidiaphragm assay and cell-based assays using cultured primary neurons. Although ciA-C2 is small in size, it occupies a large, critical surface on H_C_A1 spanning both H_CN_A and H_CC_A subdomains, which is almost as big as that recognized by the human anti-BoNT/A IgG mAb, CR1/CR2. All three CDRs and three out of four FR regions of ciA-C2 interact with H_C_A1 through a network of hydrogen bonds and a cation-π interaction. Most importantly, ciA-C2 effectively prevents BoNT/A1 from recognizing SV2 by simultaneously blocking toxin binding to the peptide and the carbohydrate moiety of SV2.

Structural analyses of the ciA-C2-H_C_A1 complex also provide valuable guidance for rational evolution of ciA-C2 to target other BoNT/A subtypes or other closely related toxins such as the newly identified BoNT/HA. Despite the high sequence identity and similar overall structures between H_C_HA and H_C_A1, BoNT/HA effectively dampens the binding of ciA-C2 and CR1/CR2 by changing selective antibody-interacting residues. We believe that understanding the structural basis of antibody-toxin recognition will inform us in this host-pathogen arms race. As a proof of concept, we showed that a single point mutation in the toxin (H_C_HA-K895N), which was designed based on the crystal structure, successfully revived ciA-C2 in term of H_C_HA binding.

As potential therapeutic agents, BoNT-neutralizing VHHs have some unique features in comparison to the conventional antibodies. First, camelid VHHs have been shown to be poorly immunogenic in animals^41^ and share a high degree of sequence identity with human V_H_, thus are expected to display low immunogenicity in human recipients. In contrast, the currently available antitoxin BAT is a mixture of equine-derived polyclonal IgG antibodies proteolytically processed to F(ab)2 to reduce immunogenicity. The immunogenicity of camelid VHHs could be further reduced by the well-developed humanization strategy^42^. Second, VHHs could be rapidly and economically produced using bacteria, a major advantage over the expensive and complicated IgG production using mammalian systems. Third, VHHs could be administered using injection, inhalation, or oral delivery to function in the gastrointestinal tract^43^, while antibodies typically need an intravenous delivery. For example, an anti-RSV (respiratory syncytial virus) VHH intended for administration through inhalation is currently in phase IIa clinical trials. Recently, a number of neutralizing VHHs against tetanus and *Clostridium difficile* toxins have also been developed^44–47^, largely motivated by the unique features of VHHs. VHHs have also been generated against other therapeutic targets, some are already in clinical trials, e.g. the anti-vWF (von Willebrand factor) VHH in phase III clinical trial (Ablynx). With their unique features added to the full antigen binding capacity and high specificity, VHHs are becoming promising components of new anti-BoNT antitoxins.

## Methods

### Study approval

All procedures using rat hippocampal neurons were conducted in accordance with the guidelines approved by the Institute Animal Care and Use Committee (IACUC) at Boston Children’s Hospital (#3030). For the mouse phrenic nerve (MPN) hemidiaphragm assay and the rat brain synaptosome binding assay, animals were sacrificed by trained personnel before dissection of organs according to §4 Abs. 3 (killing of animals for scientific purposes, German animal protection law (TSchG)). Animal number is reported yearly to the animal welfare officer of the Central Animal Laboratory and to the local authority, Veterinäramt Hannover.

### Plasmid construction

H_C_A1 (residues N872-L1296 of BoNT/A1) was cloned into expression vector pQE30 with an N-terminal His_6_-tag followed by a PreScission protease cleavage site. H_C_HA (residues E860-L1288 of BoNT/HA) was cloned into pGEX-6P-1 vector with an N-terminal glutathione S-transferase (GST) and a PreScission protease cleavage site. All point mutations for H_C_A1 and H_C_HA were generated with QuikChange site-directed mutagenesis (Agilent). Human SV2C-Loop4 (residues V473-T567) was cloned into two different vectors: pET28a vector for *E. coli* expression (bSV2C) and pcDNA vector for mammalian cell expression (gSV2C), as previously described^17^. SUMO (Saccharomyces cerevisiae S288c Smt3p) was introduced to the N-terminus of bSV2C to facilitate protein expression and folding. The VHHs ciA-C2 and ciA-F12 were cloned into the pET-32 vector downstream of the thioredoxin (Trx) tag and an enterokinase cleavage site.

### Protein expression and purification

H_C_A1, H_C_HA, bSV2C and the two VHHs were expressed in *E. coli* strain BL21-Star (DE3) (Invitrogen). Bacteria were cultured at 37 °C in LB medium containing appropriate selecting antibiotics. The temperature was set to 18 °C when OD_600_ reached 0.4. For induction, IPTG (isopropyl-b-D-thiogalactopyranoside) with a final concentration of 0.2 mM was added to the culture when OD_600_ reached 0.7. Protein expression was continued at 18 °C for 16 hours after induction. The cells were harvested by centrifugation.

Bacteria expressed proteins were first purified using Ni-NTA (Qiagen) affinity resins in a buffer containing 50 mM Tris-HCl, pH 8.0, 400 mM NaCl, and 40 mM imidazole. The bound His-tag proteins were eluted with a high-imidazole buffer (50 mM Tris-HCl, pH 8.0, 400 mM NaCl, and 300 mM imidazole) and then dialyzed at 4 °C against a buffer containing 20 mM HEPES-NaOH, pH 7.5, and 150 mM NaCl. The His-tags of H_C_A1 and H_C_HA were cleaved by PreScission protease. The Trx-tags of the VHHs were cleaved by enterokinase.

Tag-cleaved H_C_A1 and H_C_HA were further purified by MonoS ion-exchange chromatography (GE Healthcare) in a buffer containing 50 mM MES, pH 6.0, and eluted with a NaCl gradient. The peak fractions were then subjected to Superdex 200 size-exclusion chromatography (SEC, GE Healthcare) in a buffer containing 20 mM sodium phosphate, pH 6.0, and 50 mM NaCl. bSV2C and the VHHs were purified using Superdex 200 SEC in a buffer containing 20 mM HEPES-NaOH, pH 7.5, and 150 mM NaCl.

gSV2C was expressed and secreted from HEK 293 cells and purified directly from cell culture medium using Ni-NTA resins. gSV2C was eluted from the resins with a high concentration of imidazole and dialyzed against a buffer containing 50 mM Tris-HCl, pH 8.0, and 400 mM NaCl. gSV2C was then subjected to Superdex 200 SEC in a buffer containing 20 mM HEPES-NaOH, pH 7.5, and 150 mM NaCl.

### Pull-down assay

Pull-down assay was performed using Ni-NTA resins in a buffer containing 50 mM Tris-HCl, pH 8.0, 400 mM NaCl, 10 mM imidazole, and 0.1% Tween-20. bSV2C-SUMO, gSV2C, or ciA-C2 were used as baits while H_C_A1 or H_C_HA variants as preys. Protein baits were pre-incubated with Ni-NTA resins at 4 °C for 1 hour, and the unbound proteins were washed away. The resins were equally divided into small aliquots with each has ~5 μg of bound protein baits, and then H_C_A1 or H_C_HA variants (~30 μg) were added. For experiment shown in Fig. 4C, ciA-C2 (~18 μg) was used at 2-fold molar excess over H_C_A1. The pull-down assay was carried out at 4 °C for 1.5 hours. The resins were washed twice before the addition of SDS sample buffer (with 300 mM imidazole), and analyzed by SDS-PAGE. The experiment was performed in triplicates and a representative gel photo was shown. Protein band intensity was quantified by densitometry.

### Crystallization

Initial crystallization screens for the H_C_A1-ciA-C2 complex were carried out using a Phoenix crystallization robot (Art Robbins Instrument) with high-throughput crystallization screening kits (Hampton Research and Qiagen). Extensive manual optimization was then performed. Single crystals were grown at 20 °C by the hanging-drop vapor-diffusion method in a 1:1 (v/v) ratio of protein and the reservoir containing 100 mM sodium acetate, pH 5.0, and 18% Polyethylene glycol (PEG) 6,000. The crystals were cryoprotected in the original mother liquor supplemented with 20% (v/v) glycerol and flash-frozen in liquid nitrogen.

### Data collection and structure determination

X-ray diffraction studies were performed at the Advanced Photon Source (APS) and the Stanford Synchrotron Radiation Lightsource (SSRL). The best data set were collected at APS and processed with XDS^48^. The structure of the H_C_A1-ciA-C2 complex was determined with Phaser^49^ by molecular replacement using the structure of H_C_A1 (PDB: 3FUO) and ciA-F12 (PDB: 3V0A) as search models. The structural modeling and refinement were carried out iteratively using COOT^50^ and Refmac from the CCP4 suite^51^. All refinement progress was monitored with the R_free_ value with a 5% randomly selected test set^52^. The structure was validated through the MolProbity web server^53^. Data collection and structural refinement statistics are listed in Table 1. All structure figures were prepared with PyMOL (http://www.pymol.org/).

### Antibodies and toxins

Mouse monoclonal antibodies for Syb (Cl69.1), SNAP-25 (Cl71.2), SV2 (pan-SV2) were generously provided by E. Chapman (Madison, WI) and are available from Synaptic Systems (Goettingen, Germany). BoNT/A1 and rabbit polyclonal antibodies for BoNT/A1 were generously provided by E. Johnson (Madison, WI).

### Primary neuron culture, immunostaining, and immunoblot analysis

Dissociated rat hippocampal neurons were prepared from E18-19 embryos as described previously^54^. Briefly, dissected hippocampi were dissociated with papain following manufacture instructions (Worthington Biochemical, NJ). Cells were plated on poly-D-lysine coated glass coverslips and cultured in Neurobasal medium supplemented with B-27 (2%) and Glutamax (Invitrogen). Experiments were carried out using mature neurons (between 12–18 days *in vitro*). The high K^+^ buffer contains (mM: NaCl 87, KCl 56, KH_2_PO_4_ 1.5, Na_2_HPO_4_ 8, MgCl_2_ 0.5, CaCl_2_ 1). Immunostaining was carried out by fixing cells with 4% paraformaldehyde, permeabilized with 0.3% Triton in PBS solution, and incubated with indicated primary antibodies for 1 h at room temperature, followed by incubation with secondary antibodies for 1 h at room temperature. Images were collected using a confocal microscope (Leica TCS SP5; 40× oil objective). Immunoblot analysis was carried out by lysing cultured neurons with RIPA buffer (50 mM Tris-HCl, 1% NP40, 150 mM NaCl, 0.5% sodium deoxycholate, 0.1% SDS) plus a protease inhibitor cocktail (Sigma-Aldrich). Lysates were centrifuged for 10 min at maximum speed using a microcentrifuge at 4 °C. Supernatants were subjected to SDS-PAG and immunoblot analysis using the enhanced chemiluminescence (ECL) method (Pierce).

### Mouse phrenic nerve (MPN) hemidiaphragm assay

The MPN assay was performed as described previously^14, 55^. To limit the consumption of mice, the left and right phrenic nerve hemidiaphragms were excised from female mice of strain RjHan:NMRI (18–25 g, Janvier, St Berthevin Cedex, France) and placed in an organ bath containing 4 mL of Earle’s Balanced Salt Solution. The pH was adjusted to 7.4, and oxygen saturation was achieved by gassing with 95% O_2_ and 5% CO_2_. The phrenic nerve was continuously electro-stimulated at a frequency of 1 Hz via two ring electrodes. The pulse duration was 0.1 ms and the current was 25 mA to achieve maximal contraction amplitudes. Isometric contractions were recorded with a force transducer (Scaime, Annemasse, France) and the software VitroDat (Föhr Medical Instruments GmbH (FMI), Seeheim, Germany). The resting tension of the hemidiaphragm was approximately 10 mN. In each experiment, the preparation was first allowed to equilibrate for 15 min under control conditions. Then, the buffer was changed to 4 mL of Earle’s Balanced Salt Solution supplemented with 0.1% BSA and the BoNT/A1-VHH-containing solution. To determine neutralization by the VHH, a constant BoNT/A1 concentration of 1.63 pM yielding a paralysis time t_½_ of 53.7 min in the absence of VHH was incubated for 15 min at 37 °C with increasing concentrations of VHH. The previously reported calibration curve determined for BoNT/A1 (y (BoNT/A1; 0.408/0.81/1.63 pM) = −16.062Ln(x) + 60.648, R^2^ = 0.99)^56^ was used to calculate the concentration of non-neutralized BoNT/A1. Difference of applied and non-neutralized BoNT/A1 calculates the amount of neutralization.

### Binding of H_C_A1 to synaptosomes

Rat brain synaptosomes were freshly prepared and H_C_ fragment of BoNT/A1 (^35^S-H_C_A1) was radiolabeled by *in vitro* transcription/translation as previously described^14^. ^35^S-H_C_A1 was bound in the presence of 200 nM nM VHH to synaptosomes in a total volume of 100 µL of physiological buffer (140 mM NaCl, 5 mM KCl, 1 mM MgCl_2_, 1 mM CaCl_2_, 20 mM HEPES-NaOH, 10 mM glucose, 0.5% BSA, pH 7.4) with the final synaptosomal protein adjusted to a concentration of 10 mg/ml for 2 h at 4 °C. Controls were performed with samples lacking synaptosomes and/or VHH. After incubation, synaptosomes were collected by centrifugation (5,000 × g; 5 min) and unbound material in the supernatant fraction was discarded. The pellet fractions were washed two times each with 100 µL of physiological buffer and incubated for 20 min at 37 °C in SDS sample buffer and subsequently subjected to SDS-PAGE and autoradiography. Bound ^35^S-H_C_A1 was quantified with the Advanced Image Data Analyzer (AIDA 2.11) software (Raytest, Straubenhardt, Germany) and calculated after subtraction of the value obtained for control sample in the absence of synaptosomes as the percentage of the total ^35^S-H_C_A1 in the assay.

### SNAP-25 endopeptidase assay

For SNAP-25 endopeptidase assays, 10 nM BoNT/A1 was pre-incubated for 15 min at 37 °C with 30 nM VHH and subsequently added to 3 µM of rSNAP-26H6 in a total volume of 120 µL in toxin assay buffer (20 mM HEPES-KOH, pH 7.2, 150 mM K-Glu, 10 mM DTT, and 0.2 mM ZnCl_2_) at 37 °C for 1 h. Reactions were stopped by mixing with 4-fold Laemmli buffer. Samples were boiled for 2 min and subjected to 15% SDS-PAGE. Proteins were visualized by staining with Coomassie blue.

### Data Availability

Coordinates and structure factors for the H_C_A1-ciA-C2 complex have been deposited in the Protein Data Bank under accession code 5L21.

## Electronic supplementary material

Supplement
