# Supplementary material for: A camelid single-domain antibody neutralizes botulinum neurotoxin A by blocking host receptor binding
Source: Sci Rep. 2017 Aug 7;7:7438. doi: 10.1038/s41598-017-07457-5 (PMC5547058; doi:10.1038/s41598-017-07457-5)
Supplement: Supplementary file 1 — Supplement [file 41598_2017_7457_MOESM1_ESM.pdf]

Supplementary Information

A camelid single-domain antibody neutralizes botulinum neurotoxin A by blocking host receptor binding

Guorui Yao[1], Kwok-ho Lam[1], Jasmin Weisemann[2], Lisheng Peng[3], Nadja Krez[2], Kay Perry[4], Charles B. Shoemaker[5], Min Dong[3], Andreas Rummel[2], Rongsheng Jin[1*]

[1] Department of Physiology and Biophysics, University of California, Irvine, California, USA.
[2] Institut für Toxikologie, Medizinische Hochschule Hannover, Hannover, Germany.
[3] Department of Urology, Boston Children's Hospital, Department of Microbiology and Immunobiology and Department of Surgery, Harvard Medical School, Boston, Massachusetts, USA.
[4] NE-CAT and Department of Chemistry and Chemical Biology, Cornell University, Argonne National Laboratory, Argonne, Illinois USA.
[5] Department of Infectious Diseases and Global Health, Tufts Clinical and Translational Science Institute, North Grafton, Massachusetts, USA

*Correspondence to: R.J. (r.jin@uci.edu),

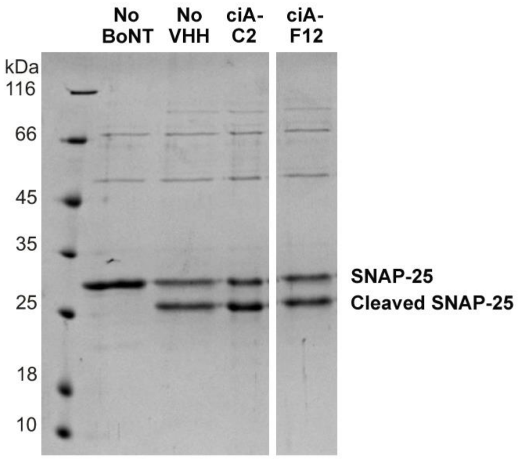

Supplementary Figure 1. VHH ciA-C2 and ciA-F12 do not affect the enzymatic activity of BoNT/A1. *In vitro* SNAP-25 endopeptidase assay where BoNT/A1 (10 nM) was pre-incubated for 15 min at 37 °C with ciA-C2 or ciA-F12 (30 nM), respectively. The BoNT/A1-VHH mixture was then added to recombinant SNAP-25 (3 µM) for 60 min at 37 °C. The lower band shows BoNT/A1-cleaved SNAP-25.

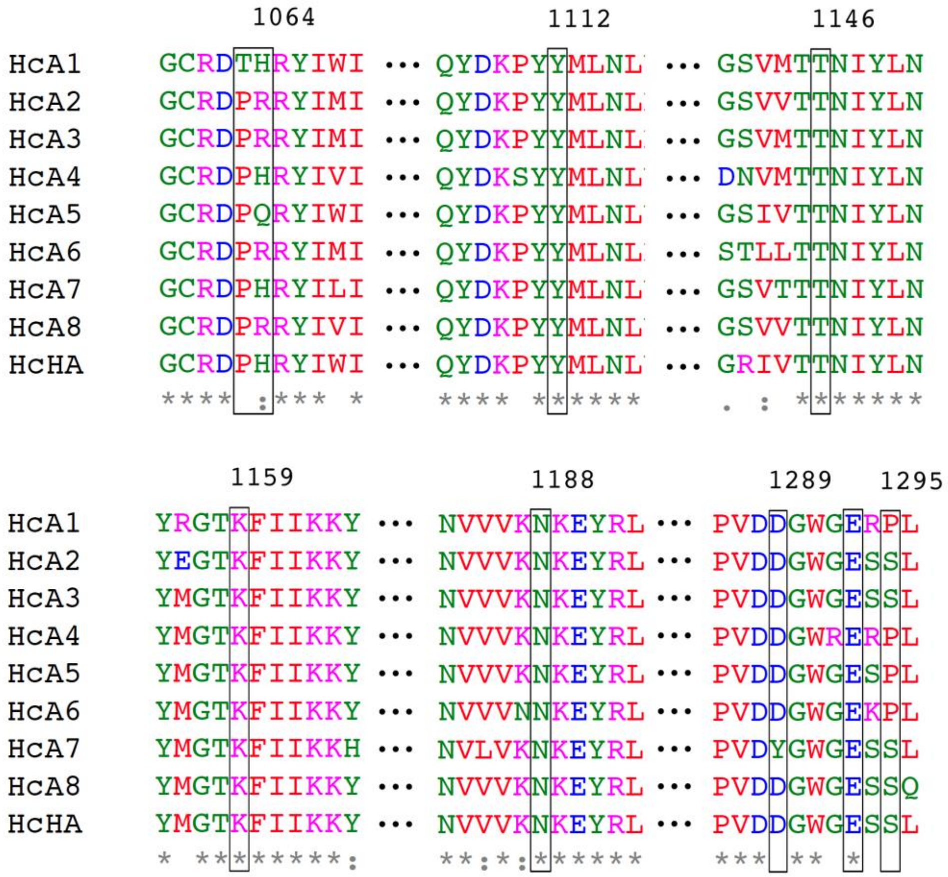

Supplementary Figure 2: The ciA-C2-interacting residues (indicated by black boxes) are largely conserved among BoNT/A1-A8 and BoNT/HA. The amino acid sequences are taken from GenBank: AAQ06331.1 (A1), ACO83782.1 (A2), ABA29017.1 (A3), ACQ51417.1 (A4), ACG50065.1 (A5), ACW83608.1 (A6), AFV13854.1 (A7), AJA05787.1 (A8), and KGO15617.1 (HA). The numbering is based on the sequence of BoNT/A1. Sequence alignment was made using Clustal Omega [1].

Supplementary Table 1: Hydrogen bonds between ciA-C2 and HCA1

Residues (ciA-C2, Chain B)	Distance (Å)	Residues (HCA1, Chain A)
Gly 26 [O]	2.71	Asn1188 [ND2]
Gly 28 [N]	2.97	Asn 1188 [OD1]
Trp 30 [NE1]	3.05	Asn 1188 [OD1]
Trp 30 [NE1]	3.37	Asn 1188 [O]
Thr 37 [OG1]	2.69	Asp 1289 [OD1]
Glu 50 [OE1]	2.99	Thr 1146 [N]
Glu 50 [OE1]	2.74	Thr 1146 [OG1]
Leu 54 [O]	2.80	His 1064 [NE2]
Thr 65 [OG1]	2.75	His 1064 [ND1]
Tyr 66 [O]	2.70	Thr 1063 [OG1]
Glu 106 [OE2]	2.53	Tyr 1112 [OH]
Trp 108 [NE1]	3.13	Glu 1293 [O]

Reference

1 Sievers, F. *et al.* Fast, scalable generation of high-quality protein multiple sequence alignments using Clustal Omega. *Mol Syst Biol* **7**, 539, doi:10.1038/msb.2011.75 (2011).
